# Are the Teachers and Students Satisfied: Sustainable Development Mode of Entrepreneurship Education in Chinese Universities?

**DOI:** 10.3389/fpsyg.2020.01738

**Published:** 2020-07-21

**Authors:** Yangjie Huang, Lanying Liu, Lanyijie An

**Affiliations:** Institute of China Innovation & Entrepreneurship Education, Wenzhou Medical University, Wenzhou, China

**Keywords:** sustainable development, entrepreneurship education, satisfaction, universities, quality, higher education, empirical study

## Abstract

Entrepreneurship education plays an important role in sustainable development. Chinese governmental agencies are making all-out efforts to promote schoolwide entrepreneurship education from top to bottom to achieve sustainable economic and social development. This is a phenomenon worth studying and summarizing. Improving the satisfaction degree of teachers and students of entrepreneurship education in universities for sustainable development becomes a typical mode of entrepreneurship education in Chinese universities. The data are derived from 12,269 valid questionnaires in student volume and 1,241 valid questionnaires in teacher volume from top universities in China, and regression and variance analyses were applied. The results show that the overall satisfaction of teachers and students is higher than the national average. There are significant differences in the overall satisfaction of different types of teachers and students. Teachers are most satisfied with the organizational leadership measures of the universities in entrepreneurship education and least satisfied with the lack of professional human resource management strategies for entrepreneurship education teachers. Students are most satisfied with entrepreneurship policy and least satisfied with entrepreneurship learning, especially that entrepreneurship theory learning and practice learning are closely combined with students’ majors. The overall satisfaction of students of entrepreneurship education mainly comes from the influence of “entrepreneurship policy dividend,” entrepreneurship learning, and entrepreneurship competition and entrepreneurship practice, which go hand in hand. The overall satisfaction of teachers is most affected by the satisfaction of organizational leadership, followed by the satisfaction of mechanism guarantee and teaching management. This study introduces the sustainable development model of entrepreneurship education in top Chinese universities through the improvement of satisfaction of entrepreneurship education and has certain reference significance for managers and teachers of entrepreneurship education practice in other developing countries.

## Introduction

Entrepreneurship education is growing worldwide (Kuratko, 2005; Siegel et al., 2007; Katz, 2008). Innovation and entrepreneurship are also seen as a new engine of China’s economic growth (Li et al., 2003). After more than 20 years of development, entrepreneurship education in Chinese universities is unprecedented in both quantity and scale (Weiming et al., 2016). The sustainable development of entrepreneurship education has become an important theme (Wyness and Sterling, 2015; Wyness et al., 2015). Satisfaction is an important tool to measure the quality of education (Tan and Kek, 2004; Gruber et al., 2010). The literature on sustainability within the entrepreneurship discipline remains extremely limited (Wyness et al., 2015). Some studies have shown a strong relationship between satisfaction and sustainability (Zairi, 2002; Ogbari and Borishade, 2015). Furthermore, how to improve the satisfaction degree of teachers and students of entrepreneurship education in universities for the sustainable development is an important research topic.

Satisfaction studies are applied to a wide range of fields. Such as academic job satisfaction (Shin and Jung, 2014), university satisfaction (Bowman and Smedley, 2013), career satisfaction (Lounsbury et al., 2012), student satisfaction (Douglas et al., 2015; Wang et al., 2019), and teachers’ job satisfaction (Neto et al., 2017). In the field of entrepreneurship, Noorderhaven et al. (2004) found that dissatisfaction at the level of societies has a positive and significant influence on self-employment levels. Entrepreneurial job characteristics of autonomy, diversity, and feedback are significant predictors of entrepreneurial job satisfaction (Schjoedt, 2009). Family members’ satisfaction with business performance is a better indicator of business performance (Mahto et al., 2010). Furthermore, the satisfaction degree survey of teachers and students is an important tool to improve the sustainable development of higher education. The satisfaction of teachers and students on entrepreneurship education is also an effective tool to evaluate the quality of entrepreneurship education in universities for sustainable development.

Application of satisfaction in entrepreneurship education has just begun to receive scholarly attention (Matlay and Carey, 2007; Wu and Song, 2019). Therefore, our research questions were as follows: Are the teachers and students satisfied in Chinese universities for the sustainable development of entrepreneurship education? What are the differences between the satisfaction of teachers and students with different types of characteristics? What factors affect the satisfaction of teachers and students on entrepreneurship education in universities?

## Literature Review

Synthesizing this fast-growing body of empirical research and reviews on entrepreneurship education suggests two main patterns. First, a large number of studies on the impact of entrepreneurship education have focused on entrepreneurial attitudes and intentions (Nabi et al., 2017), for instance, entrepreneurial intentions of business and engineering students (Voda and Florea, 2019), entrepreneurial intentions of science and engineering students in China (Fang and Chen, 2019), and internship quality on entrepreneurial intentions (Yi, 2018). Second, few reviews focus on entrepreneurship education satisfaction of teachers and students specifically in higher education for sustainable development. These two distinct yet related research gaps form the rationale for this article.

### Entrepreneurship Education in Universities in China

Entrepreneurship education fosters entrepreneurial attitudes, skills, and mindset (Fayolle and Gailly, 2008). The principal role of entrepreneurship education is to promote students’ entrepreneurial intentions and increase their awareness that the entrepreneurial path is a viable career option (Liu F. et al., 2019). Fayolle et al. (2019) offered new and innovative conceptual frameworks to bridge research and practitioner gaps in entrepreneurship education theory and practice. Some of their studies showed that (1) the effects vary depending on trainees’ personal characteristics; (2) training strategies can have different impacts on learning processes and results; (3) the environment and the social context might foster or hinder training results; and so on (Fayolle et al., 2013, 2019). There were four modes of nature of EE pedagogical methods: the supply model focusing on reproduction methods; demand model focusing on personalized/participative methods; competence model focusing on communication, discussion, and production methods; and hybrid models (Nabi et al., 2017). Furthermore, the teacher educators used a relatively large number of the pedagogical models and methods pursued in entrepreneurship education, such as problem-based learning and experiential and practical descriptions of situations (Seikkula-Leino et al., 2015). These studies provide very good references for China’s entrepreneurship education research.

In the report of the 19th National Congress of the Communist Party of China in October 2017 (Jinping, 2017), President Xi Jinping pointed out that the construction of world-class universities and disciplines should be accelerated. Innovation and entrepreneurship education should run through the whole process of talent training. In 2017, there were 2,631 institutions of higher learning, with a total of 37.79 million students in higher education. The gross enrollment rate of higher education reached 45.7%. A new round of scientific and technological revolution and industrial transformation is sweeping across the world, and China’s accelerated transformation of its economic development model is forming a historic intersection. The country’s innovative development and industrial upgrading have an unprecedented urgent demand for talents. Both the logic of education internal development and the logic of national development put forward new and higher requirements for higher education reform and innovation.

“China’s education modernization 2035” puts forward eight basic concepts for promoting education modernization: pay more attention to putting morality first, pay more attention to all-round development, pay more attention to serving all people, pay more attention to lifelong learning, pay more attention to teaching students in accordance with their aptitude, pay more attention to the unity of knowledge and practice, pay more attention to integrated development, and pay more attention to joint construction and shared benefits. Certainly, serving people and making people satisfied are the fundamental principles of education. In addition, to improve the satisfaction degree of teachers and students of entrepreneurship education in universities for sustainable development becomes a typical mode of entrepreneurship education in Chinese universities.

In September 2018, the State Council issued opinions on promoting high-quality development of innovation and entrepreneurship to create an upgraded version of mass entrepreneurship and innovation. China is making a strategic transition from employment education to innovation and entrepreneurship education (Yu, 2018). China’s concept of mass entrepreneurship and innovation has been written into the United Nations (UN) resolution, and the entrepreneurship rate of graduates has exceeded 3%. China’s “Internet+” contest of college students’ innovative undertaking was personally proposed by prime minister Li Keqiang; since 2015, there have been four competitions in total, with 4.9 million college students and 1.19 million teams, which led to the emergence of a large number of high-quality project with considerable good social benefits for the sustainable development of the whole country.

### Sustainable Development and Entrepreneurship Education

The term *sustainable development* was first coined at the UN Conference on the Human Environment in 1972 and later gained prominence by way of a report to the UN by the World Commission on Environment and Development (Colvin et al., 2014). As we know, entrepreneurship is increasingly recognized as the transformation to sustainable products and processes, with many prominent thinkers advocating entrepreneurship as a panacea for many social and environmental problems.

It is undeniable that entrepreneurship education plays an important role in sustainable development (Wyness et al., 2015). There are many examples of sustainable development through entrepreneurship education. Entrepreneurial university embarking on the path of sustainable development goals (SDGs) requires HEI to design, launch, implement, and customize specific process architecture to govern the advance of the sustainability approach (Fleaca et al., 2018). Chinese governmental agencies are making all-out efforts to promote schoolwide entrepreneurship education from top to bottom to achieve sustainable economic and social development. This is a phenomenon worth studying and summarizing.

### Satisfaction of Entrepreneurship Education

Entrepreneurship education programmers create high job satisfaction and enhance life status (Din et al., 2016). Individuals who are concerned about further management education and entrepreneurship education show themselves to be more innovative. Moreover, indirectly, by means of the relationship between innovation and success, specific entrepreneurship education contributes to obtaining better business results (Cruz Natalia et al., 2009).

There are many ways to evaluate the quality of entrepreneurship education in universities, such as performance excellence management (Cao and Jiang, 2017), the principle of brain neurology (Li, 2018), linguistic operators (Yuan et al., 2018), and priority-degree evaluation model (Zhou, 2018). Nevertheless, the satisfaction of teachers and students of entrepreneurship education still is an effective and important tool to evaluate the quality of entrepreneurship education; in addition, it is also easy to carry out a large-sample survey.

Satisfaction studies originated from customer satisfaction studies in Europe and the United States. In the 1960s, American scholar Juillerat put forward the measurement scale of student satisfaction and also established a special satisfaction measurement company (Juillerat and Schreiner, 1996). There is a direct relationship between academic satisfaction and professional identity (Santisi et al., 2018). Peg transfer time, knot tying time, satisfaction with performance, and post-self-efficacy were dependent variables of skill acquisition of medical students (Dempsey and Kauffman, 2017). Malinen and Savolainen (2016) analyzed the effectiveness of entrepreneurship education and whether it had a positive effect on job satisfaction.

However, less literature has focused on the satisfaction of entrepreneurship education. The educational satisfaction of teachers and students refers to the complex subjective experience of teachers and students when they compare their perceived educational opportunities, educational processes, and educational results with their own expectations, investments, and historical development levels (Wadsworth et al., 2008). Teachers and students’ satisfaction of entrepreneurship education should include overall satisfaction and sub-satisfaction; overall satisfaction is the quality of entrepreneurship education in colleges and universities that is directly perceived and subjectively experienced as a whole by students and teachers. Student sub-satisfaction includes the policy on entrepreneurship education, entrepreneurship education learning, entrepreneurship education competition, and entrepreneurship education practice. Teacher sub-satisfaction refers to the perception and subjective experience of policy mechanisms or educational activities such as the curriculum system, organizational leadership, teacher construction, teaching management, and mechanism guarantee.

### Factors Influencing the Satisfaction of Entrepreneurship Education

Entrepreneurship educational quality influences student satisfaction, and the contents and methods of education are important factors in the evaluation of entrepreneurship education quality (Sigala et al., 2006). For example, the teaching staff, the teaching methods, and course administration are key elements to achieving student satisfaction and their subsequent loyalty to ensure the university’s survival (Marzo Navarro et al., 2005). Family support did not have a moderating effect on entrepreneurship and entrepreneurial education satisfaction on entrepreneurial intention (Jo, 2019).

With the continuous promotion of entrepreneurship education reform in colleges and universities, domestic scholars have begun to explore the relevant influencing factors or mechanisms of the satisfaction of teachers and students in entrepreneurship education in colleges and universities in China from different perspectives (Zhou and Xu, 2012). For example, the gender difference of Tsinghua University students, professional status, whether students are from single-child families, entrepreneurship competition and awards, entrepreneurial activity experience, and occupational factors such as parents will affect the students’ entrepreneurial attitude and entrepreneurial tendencies (Baoshan and Depeng, 2017). Some Chinese scholars used the structural equation model and explored the influence of entrepreneurial education satisfaction on entrepreneurial behavior in Chinese universities and put forward six dimensions of entrepreneurial education satisfaction, namely, satisfaction with the course system, satisfaction with practice, satisfaction with the faculty system, satisfaction with teaching methods, satisfaction with teaching departments, and satisfaction with teaching objectives. Other scholars analyzed the influence of entrepreneurship education in colleges and universities from four aspects, entrepreneurship courses, entrepreneurship lectures, entrepreneurship competitions, and entrepreneurship societies, and also found that entrepreneurship education policies, entrepreneurship education environment, family entrepreneurship background, and entrepreneurship education content have a significant impact on college students’ satisfaction.

### Conceptual Models and Hypotheses

According to the above literature review, there are two deficiencies in existing studies: (1) in terms of research content, there is almost no research on the satisfaction degree of entrepreneurship education from the dual perspectives of teachers and students in universities, which is in sharp contrast to the government’s efforts to promote the high-quality development of innovation and entrepreneurship and create an upgraded version of “mass entrepreneurship and innovation”; (2) on the research methods, although most have used quantitative empirical methods, the satisfaction measurement scale for this study provides a lot of references. The sample size of existing studies is generally small. The relationship between different entrepreneurial education contents and satisfaction is also less studied. The following six hypotheses are formulated.

**H1:** There are significant differences between teachers and students in different types of colleges and universities in their overall satisfaction with the quality of entrepreneurship education.

**FIGURE 1:**
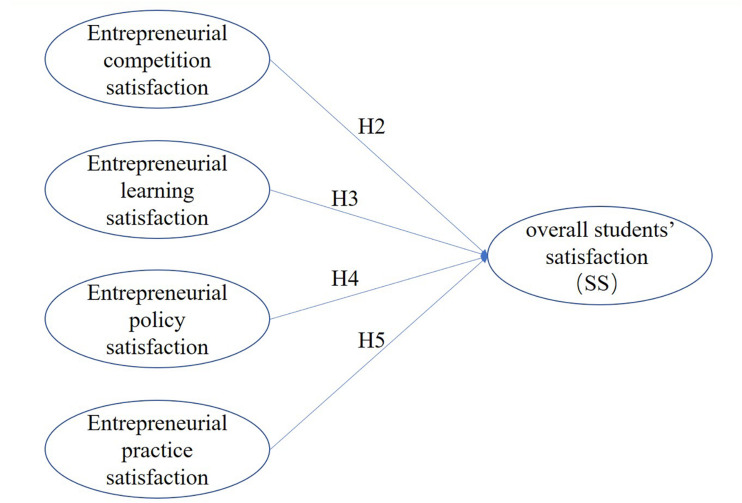
Research framework of SS.

Furthermore, Figure 1 shows the hypothesized structural model for the possible influence of the itemized satisfaction on students’ overall satisfaction with the quality of entrepreneurship education (SS). After the preliminary investigation of the current status among 200 students in Zhejiang province, the following four hypotheses are formulated.

**H2:** Entrepreneurial competition satisfaction has a positive effect on SS.

**H3:** Entrepreneurial learning satisfaction has a positive effect on SS.

**H4:** Entrepreneurial policy satisfaction has a positive effect on SS.

**H5:** Entrepreneurial practice satisfaction has a positive effect on SS.

In addition, we studied the possible influence of the itemized satisfaction (see Table 1 and 2) on teachers’ overall satisfaction with the quality of entrepreneurship education (TS). After the preliminary investigation of the current status among 100 teachers in Zhejiang province, the following hypothesis is formulated.

**TABLE 1:** The sample composition of teachers and students.

Basic characteristics	Grouping	Capita	Percentage
**Teacher volume (*N* = 1,241)**			
Gender	Male	660	53.2
	Female	581	46.8
Age	30 years old and under	410	33.0
	31–35 years old	319	25.7
	36–40 years old	333	26.8
	41 years old and above	179	14.4
Work experience related to entrepreneurship education	Within 2 years	406	32.7
	3–5 years	299	24.1
	6–9 years	250	20.1
	10 years and above	286	23.0
**Student volume (*N* = 12,269)**			
Gender	Male	5,571	45.4
	Female	6,698	54.6
Single-child family	Yes	6,512	53.1
	No	5,757	46.9
Entrepreneurial practice experience	Yes	2,729	22.2
	No	9,540	77.8
Registered permanent residence	Cities and towns	6,613	53.9
	Country	5,656	46.1
Academic achievement	Top 25%	4,638	37.8
	Above average 25%	4,009	32.7
	Below average 25%	2,578	21.0
	Low 25%	1,044	8.5

**TABLE 2:** The overall satisfaction frequency of teachers and students on the quality of entrepreneurship education.

Score values	Frequency		Percentage		Cumulative percentage	
	Teacher	Student	Teacher	Student	Teacher	Student
1	21	274	1.7	2.2	1.7	2.2
2	56	641	4.5	5.2	6.2	7.5
3	297	3,958	23.9	32.3	30.1	39.7
4	551	4,600	44.4	37.5	74.5	77.2
5	316	2,796	25.5	22.8	100.0	100.0
Total	1,241	12,269	100.0	100.0

**H6:** The school’s measures on curriculum and organizational construction have a positive impact on TS.

## Materials and Methods

### Data Collection and Procedure

While a large amount of research has focused on entrepreneurial intentions, very few studies have examined the satisfaction of the quality of entrepreneurship education (Hsiung, 2018). As many existing surveys are poorly designed, lack standardization, and give no evidence concerning reliability or validity, Gruber et al. (2010) used a new tool to measure 15 dimensions of student satisfaction at an institutional level. This study was decided to develop a new measurement tool, in addition, that combines students and teachers’ perspectives in the satisfaction degree of entrepreneurship education in top universities. This study used a quantitative research method because it explains the causes of changes in social factors, primarily through objective measurement and quantitative analysis (Yi, 2018).

The questionnaire has been widely used in the research on the influence of entrepreneurship education (Onuma, 2016; Binti Othman and Othman, 2017; Asghar et al., 2019). On September 15, 2018, to January 18, 2019, through questionnaires (via IP restrictions, questionnaires were limited to a device, such as mobile phones, computers, and others, which were restricted to only one answered questionnaire) disseminated across the whole country, 1,231 universities in 31 provinces (autonomous regions and municipalities directly under the central government) were surveyed, resulting to 201,034 records. Specific details are as follows: a total of 1,231 universities were surveyed in the “student” questionnaire, involving 31 provinces (autonomous regions and municipalities directly under the central government), and 170,764 valid questionnaires were obtained, accounting for 90.87%. A total of 596 universities were surveyed in the questionnaire of “teacher,” and 12,596 effective questionnaires were obtained, accounting for 96.01%. The survey objects and requirements of students are as follows: undergraduate students (except freshmen of class 2018) who have entrepreneurship education experience and undergraduate students who have graduated in the past 5 years, excluding postgraduate students and doctoral students. The survey objects and requirements of teachers are as follows: leading cadres, administrative personnel, and professional teachers related to entrepreneurship education. In this paper, the types of universities are mainly selected as “double world-class universities,” with 12,269 valid questionnaires in student volume and 1,241 valid questionnaires in teacher volume (see Table 1).

### Scale Reliability and Validity Test

The teacher–student measurement scale used in this project is based on the domestic and foreign journal literature (Zhou and Xu, 2012), compared and analyzed the existing questionnaire on entrepreneurship education (Asghar et al., 2019), and combined with the in-depth semi-structured interview analysis of several experienced entrepreneurship education teachers (Galletta, 2013). Its design has been verified, modified, and improved many times. Literature support of indicators is shown in Tables 3, 4. In order to fully grasp the real situation of entrepreneurship education in China’s universities, the survey was conducted anonymously, and all data were only used for academic research. Therefore, according to the overall satisfaction survey, the research group designed a 5-point Likert scale measuring “your overall satisfaction on the quality of entrepreneurship education in your university” and other items for teachers and students. At the same time, in the student volume, this paper focuses on the selection of satisfaction degree of entrepreneurship policy, satisfaction degree of entrepreneurship competition, satisfaction degree of entrepreneurship learning, and satisfaction degree of entrepreneurship practice. In the teacher volume, this paper focuses on the selection of five sub-satisfaction indicators, namely, satisfaction with the curriculum system, satisfaction with organizational leadership, satisfaction with teacher construction, satisfaction with teaching management, and satisfaction with mechanism guarantee, as well as a total of 25 sub-satisfaction indicators.

**TABLE 3:** Itemized satisfaction of teachers of entrepreneurship education in “double world-class” universities.

Itemized satisfaction	The secondary indicators	Mean value	Standard deviation	Literature support of indicators
Curriculum system satisfaction (3.82)	T1 Compiled textbooks on entrepreneurship to meet students’ diverse learning needs	3.73	1.011	James, 2014; O’Rafferty et al., 2014; Arranz et al., 2017
	T2 A stratified and classified curriculum system for entrepreneurship education has been established	3.82	0.998	
	T3 Open online courses such as MOOCs for entrepreneurship and case database have been established	3.85	0.965	
	T4 Flexible mechanism for mutual recognition of entrepreneurship credits	3.82	0.975	
	T5 Specialized courses for entrepreneurship education	3.87	0.973	
Organizational leadership satisfaction (3.92)	T6 Establish a special entrepreneurship management department (such as entrepreneurship academy)	3.92	1.024	Lee et al., 2015; Muralidharan and Pathak, 2018
	T7 Equipped with entrepreneurship education teachers and full-time management personnel	3.94	0.985	
	T8 Equipped with special office, practice space, and soft environment	3.94	1.008	
	T9 Attach importance to entrepreneurship education and set up the relevant work leading group	4.07	0.888	
	T10 The assessment of secondary academies includes performance indicators of entrepreneurship education	3.75	1.006	
Teacher construction satisfaction (3.80)	T11 The performance of individual entrepreneurship education is included in the performance evaluation standard of teachers	3.69	1.044	Ruskovaara et al., 2015; Neto et al., 2017
	T12 The performance of individual entrepreneurship education is included in the evaluation and employment conditions of teachers’ professional titles	3.68	1.055	
	T13 Strengthen the building of teachers’ teaching capacity in entrepreneurship education	3.89	0.931	
	T14 Entrepreneurship education teaching research projects	3.86	0.971	
	T15 Organize teachers to participate in various entrepreneurship mentor cultivation projects outside the school	3.88	0.927	
Teaching management satisfaction (3.95)	T16 Entrepreneurship education is open to all students	3.97	0.934	Birdthistle et al., 2016; Ehrlin et al., 2016
	T17 Education courses on innovation and entrepreneurship are offered to all students	3.99	0.913	
	T18 Teachers and students are encouraged to collaborate on experiments, papers, and patents	3.97	0.912	
	T19 There are policies to encourage teachers and students to work together on research and entrepreneurship projects	3.94	0.905	
	T20 Establish a school-enterprise collaborative entrepreneurship education mechanism	3.90	0.927	
Mechanism guarantee satisfaction (3.86)	T21 Actively implement policies introduced by governments at all levels to support business startups	3.96	0.891	Ribeiro-Soriano and Galindo-Martin, 2012; Wonglimpiyarat, 2013
	T22 There is an independent professional title promotion mechanism for entrepreneurship teachers	3.66	1.078	
	T23 Sufficient funds for entrepreneurship education	3.86	0.943	
	T24 Incentive mechanisms for professional teachers to participate in entrepreneurship education and teaching	3.87	0.946	
	T25 Encourage entrepreneurship based on cutting-edge technology entrepreneurship	3.94	0.910	

**TABLE 4:** The satisfaction degree of students’ entrepreneurship education in universities with “double world-class” construction.

Itemized satisfaction	The secondary indicators	Mean value	Standard deviation	Literature support of indicators
Entrepreneurial policy satisfaction (3.78)	S1 Entrepreneurship policy is of practical help to start a business	3.83	0.901	Zerbinati and Souitaris, 2005; Desrochers and Sautet, 2008
	S2 Entrepreneurship policies help to increase the willingness of individuals to start businesses	3.83	0.910	
	S3 Local governments have simplified the application process for college students to apply for business registration	3.74	0.930	
	S4 Start-up fund provided by the school (interest-free loan)	3.71	0.953	
Entrepreneurial competition satisfaction (3.74)	S5 Entrepreneurship competition improves entrepreneurial ability	3.66	0.966	Bolli and Woerter, 2013; Falck and Woessmann, 2013
	S6 Entrepreneurship competition boosts entrepreneurial confidence	3.69	0.961	
	S7 Entrepreneurship competitions help expand social networks	3.80	0.937	
	S8 Entrepreneurship competition promotes the team’s cooperation ability	3.88	0.917	
	S9 Entrepreneurship competition for real entrepreneurship has greater help	3.69	0.957	
Entrepreneurial learning satisfaction (3.45)	S10 There are various types of entrepreneurship education courses	3.45	1.018	Hosseini and Pouratashi, 2011; Wiley and Berry, 2015
	S11 Teachers have rich experience in entrepreneurship education and teaching	3.53	1.015	
	S12 Entrepreneurship course content and their professional knowledge are closely combined	3.29	1.054	
	S13 Entrepreneurship course content are closely combined with cutting-edge trends	3.54	0.999	
Entrepreneurial practice satisfaction (3.60)	S14 The school provides integrated entrepreneurial practice services	3.61	0.970	Greefs, 1998; dos Santos and Spann, 2011; Zou and Zhao, 2014
	S15 Entrepreneurial practice has an independent college student business park	3.67	1.006	
	S16 Entrepreneurial practice has a special off-campus practice base	3.57	0.999	
	S17 Entrepreneurial practice projects and professional learning have high integration	3.55	0.989	

The method of reliability test was to calculate the corrected item–total correlation (CITC) of each measurement item (Zijlmans et al., 2019). At the same time, the Cronbach’s alpha coefficient, which was the most widely used method for estimating internal consistency reliability (Trizano-Hermosilla and Alvarado, 2016), was calculated. If the alpha coefficient is greater than 0.7, the reliability of the index is acceptable (Santos, 1999). The method of validity test was exploratory factor analysis, KMO, and Bartlett test, and the results showed that KMO was greater than 0.7. The significance probability of the Bartlett sphere test was all 0.000, indicating that the data were correlated, that is, suitable for factor analysis (Conway and Huffcutt, 2003). All the measurement indexes used in this paper have passed the test.

## Results

### Overall Satisfaction

According to the statistics of 1,241 samples of teachers in entrepreneurship education, the overall satisfaction of teachers from “double world-class” Chinese universities is 3.87, and the satisfaction rate (i.e., the sum of four and five points) is 69.9%. The overall satisfaction score of 12,596 teachers of entrepreneurship education nationwide was 3.71, with a satisfaction rate of 59.1%. The overall satisfaction of teachers in “double world-class” universities is about 10% points higher than the national average.

Students from “double world-class” Chinese universities (*N* = 12269) scored 3.73 on the overall satisfaction with the quality of entrepreneurship education, with a satisfaction rate of 60.3%. The overall satisfaction score of 170,764 sample data of students nationwide is 3.67, and the satisfaction rate is 54.6%. The overall satisfaction of students in “double world-class” universities is about 5% points higher than the national average.

### Analysis of Difference

According to the analysis of variance (Scheffe, 1999), under the significance level of 0.05, the overall satisfaction degree of teachers and students in double world-class universities of entrepreneurship education is significantly higher than that of teachers and students in ordinary undergraduate colleges, higher vocational colleges, and independent colleges.

With gender, age, degree, and title of teachers in “double world-class” universities as variables, the overall satisfaction of teachers with the quality of entrepreneurship education is the dependent variable, and the variance analysis below the significance level of 0.05 shows the following: (1) male teachers’ satisfaction was significantly higher than that of female teachers (*F* = 4.614, *p* < 0.05); (2) there was no significant difference in satisfaction among teachers of different age groups, degrees, and titles; and (3) the overall satisfaction of teachers with more than 10 years of experience in entrepreneurship education is significantly lower than that of teachers with other working years.

In the “double world-class” universities, gender, nationality, family, major, and academic performance were taken as variables, and the variance analysis below the 0.05 significance level showed the following. (1) Male and female students had no significant difference in their satisfaction with the quality of entrepreneurship education (*F* = 2.734, *p* = 0.098). This is different from the result showing that male students in the national sample of 170,764 had significantly higher satisfaction than did female students. (2) There is no significant difference in whether students are from a single-child family. (3) Students whose parents had started a business were significantly more satisfied than those whose parents had not (*F* = 28.375, *p* < 0.001). (4) The average satisfaction degree of the student in cities was significantly higher than that of rural students (*F* = 4.825, *p* < 0.05). (5) The overall satisfaction degree of students who had entrepreneurial practice in school was significantly higher than that of students who had no entrepreneurial practice (*F* = 68.052, *p* < 0.001). (6) There was a significant difference in overall satisfaction among students of different majors, with the highest degree in engineering (3.81) and the lowest degree in education (3.54). (7) The satisfaction degree of students with good academic performance is significantly higher than that of students with poor academic performance; that is, the higher the academic performance is, the higher is the overall satisfaction degree.

### Cause Analysis of Specific Differences

#### Analysis of the Most Satisfied and Unsatisfied Indicators of Teachers in “Double World-Class” Universities

Based on the five dimensions of entrepreneurship education process from the perspective of teachers that were explored and constructed by this research group, the statistical results of further optimized selection indexes are shown in Table 3. What the teachers in double world-class universities are most satisfied with are the organizational and leadership measures like “T9 Attaches great importance to entrepreneurship education and the establishment of relevant work leading group,” as well as the university-wide entrepreneurship education of T16 and T17 and the teaching management of “T18 To encourage teachers and students to cooperate in scientific research or entrepreneurship.” The most unsatisfactory is the lack of professional human resource management strategies for entrepreneurship education teachers, including “T11 Performance appraisal,” “T12 The performance of individual entrepreneurship education is included in the evaluation and employment conditions of teachers’ professional titles,” and “T22 There is an independent professional title promotion mechanism for entrepreneurship teachers.” In terms of the entrepreneurship curriculum system, teachers from double world-class universities believe that the current entrepreneurship textbooks cannot meet students’ diverse learning needs (T1) and are in urgent need of improvement.

#### Analysis of the Most Satisfied and Unsatisfied Indicators of Students in “Double World-Class” Universities

The top five indicators with which students from double world-class universities are most satisfied as regards entrepreneurship education are “S8 Entrepreneurship competitions promote the team cooperation ability,” “S1 Entrepreneurship policy is of practical help to start a business,” “S2 Entrepreneurship policies help to increase the willingness of individuals to start businesses,” “S7 Entrepreneurship competitions help expand social networks,” and “S3 Local governments have simplified the application process for college students to apply for business registration.” In terms of specific satisfaction items, entrepreneurship policies were the most satisfying (see Table 4). In particular, driven by government policies, various national-level and provincial-level entrepreneurship competitions have greatly exercised students’ entrepreneurial teamwork ability, expanded their interpersonal network, and enhanced their confidence and comprehensive ability to start their own businesses, which have been well received by students.

The five most unsatisfied indicators (scored from low to high) of students’ entrepreneurship education in their universities are “S12 Entrepreneurship course content and their professional knowledge are closely combined,” “S10 There are various types of entrepreneurship education courses,” “S11 Teachers have rich experience in entrepreneurship education and teaching,” “S13 Entrepreneurship course content is closely combined with cutting-edge trends,” and “S17 Entrepreneurial practice projects and professional learning have high integration.” On the specific satisfaction item, the least satisfied is entrepreneurship learning (3.45). It can be seen that entrepreneurship theory learning and practice learning are closely combined with students’ majors and that carrying out academic-based entrepreneurship is an important way to improve students’ satisfaction with entrepreneurship education quality in double world-class universities.

#### Analysis of Influencing Factors on the Overall Satisfaction Degree of Students in “Double World-Class” Universities

Following the “learner-centered” philosophy (Huba and Freed, 2000), the overall satisfaction score of students in double world-class universities was further selected as the dependent variable. Based on the conceptual framework above, we assumed that entrepreneurship policy, entrepreneurship competition, entrepreneurship learning, and entrepreneurship practice in Table 4 were the influencing factors. For the entrepreneurial policy dimension, the Cronbach’s alpha coefficient is 0.911, the CITC minimum value is 0.784, the exploratory factor analysis KMO is 0.807, and the significance probability of the Bartlett sphere test is 0.000. For the entrepreneurship competition dimension, the Cronbach’s alpha coefficient is 0.932, the CITC minimum value is 0.792, the exploratory factor analysis KMO is 0.896, and the significance probability of the Bartlett sphere test is 0.000. For the entrepreneurial learning dimension, the Cronbach’s alpha coefficient is 0.891, the CITC minimum value is 0.744, the exploratory factor analysis KMO is 0.835, and the significance probability of the Bartlett sphere test is 0.000. For the entrepreneurial practice dimension, the Cronbach’s alpha coefficient is 0.918, the CITC minimum value is 0.797, the exploratory factor analysis KMO is 0.855, and the significance probability of the Bartlett sphere test is 0.000. These indicate that the reliability and validity of the independent variables are good.

After exploratory factor analysis and principal component analysis (Wold et al., 1987), the Caesar normalized maximum variance method rotated and extracted the corresponding four common factors, and the total variance interpretation degree reached 78.9%. After correlation analysis, multiple regression (Aiken et al., 1991) results of dependent variables and independent variables are shown in Table 5. The multiple correlation coefficient *R* is 0.794, the square of the multiple correlation coefficient is 0.63, and the *F* value is 5,222.54, *p* < 0.001. Since multiple linear regression is mainly adopted, in order to get a more scientific conclusion, it is necessary to test whether the regression model has three major problems, namely, multicollinearity, sequence correlation, and heteroscedasticity (Draper and Smith, 1998; Liu X. et al., 2019). The multicollinearity problem of this model is mainly tested by the variance inflation factor (VIF), which shows that there is basically no multicollinearity problem in this model (Akinwande et al., 2015). The DW value of 2.01 indicates that there is almost no sequence correlation. By observing whether the scatter plot of standardized residuals has an obvious change rule, it is also found that there is no heteroscedasticity problem (Muller and Stadtmuller, 1987). That is to say, the overall satisfaction of students in double world-class universities is most affected by the satisfaction of entrepreneurship policy, followed by entrepreneurship competition, entrepreneurship practice, and entrepreneurship learning, all of which are indispensable, and the degree of influence of entrepreneurship competition, entrepreneurship practice, and entrepreneurship learning on the overall satisfaction of students is close.

**TABLE 5:** Summary of regression analysis on the overall satisfaction of students in “double world-class” universities.

Predictor variable	Model			
	β	Standard error	*T*-value	VIF
(Constant)		0.005	0.000	
Entrepreneurial competition satisfaction	0.368	0.005	66.925***	1
Entrepreneurial learning satisfaction	0.351	0.005	63.830***	1
Entrepreneurial policy satisfaction	0.488	0.005	88.827***	1
Entrepreneurial practice satisfaction	0.366	0.005	66.684***	1
Regression model	DW	2.01		
	*F* value	5,222.54***		
	*R*^2^	0.63		

Dependent variable: overall students’ satisfaction. *** indicate significance at the levels of 5, 1, and 0.1%, respectively.

#### Analysis of Influencing Factors on the Overall Satisfaction of Teachers in “Double World-Class” Universities

Similarly, the overall satisfaction degree of teachers is taken as the dependent variable and the five dimensions in Table 3 as the independent variable. After the same correlation analysis and corresponding tests as in the student satisfaction analysis, the final regression analysis results are shown in Table 6. The multiple correlation coefficient *R* of the regression model of teachers’ overall satisfaction is 0.656, the square of the multiple correlation coefficient is 0.431, and the *F* value is 186.788, *p* < 0.001. Since multiple linear regression is also adopted, in order to ensure a more scientific conclusion, the test results show that the regression model does not have three major problems, namely, multicollinearity, sequence correlation, and heteroscedasticity. That is to say, the overall satisfaction of teachers in double world-class universities is most influenced by the satisfaction of organizational leadership (*β* = 0.289, *p* < 0.001), followed by the satisfaction of mechanism guarantee and teaching management. However, there was no significant regression in the satisfaction of the curriculum system and teacher construction.

**TABLE 6:** Summary of regression analysis on overall satisfaction of teachers in “double world-class” universities.

Predictor variable	Model			
	β	Standard error	*T*-value	VIF
(Constant)		0.021	0.000	
Curriculum system satisfaction	0.082	0.046	1.773	4.624
Organizational leadership satisfaction	0.289	0.038	7.665***	3.073
Teacher construction satisfaction	0.063	0.042	1.481	3.892
Teaching management satisfaction	0.086	0.042	2.047*	3.804
Mechanism guarantee satisfaction	0.198	0.045	4.379***	4.428
Regression model	DW	1.96		
	*F* value	186.788***		
	*R*^2^	0.431		

Dependent variable: overall teachers’ satisfaction. *, *** indicate significance at the levels of 5, 1, and 0.1%, respectively.

## Discussion

The purpose of this study was to better understand the sustainable development model of entrepreneurship education in top Chinese universities through the improvement of satisfaction of entrepreneurship education. We asked three questions: Are the teachers and students satisfied in Chinese universities for the sustainable development of entrepreneurship education? What are the differences between the satisfaction of teachers and students with different types of characteristics? What factors will affect the satisfaction of teachers and students on entrepreneurship education in universities?

Our results show the following. First, in terms of overall satisfaction with the quality of entrepreneurship education, the overall satisfaction of teachers and students in double world-class universities is the highest, among which the overall satisfaction of teachers in double world-class universities is about 10% points higher than the national average and the overall satisfaction of students in double world-class universities is about 5% points higher than the national average. Second, in terms of the difference in satisfaction with the quality of entrepreneurship education, (1) the overall satisfaction with the quality of entrepreneurship education of teachers and students in double world-class universities is significantly higher than that of teachers and students in ordinary undergraduate colleges, higher vocational colleges, independent colleges, and other schools; (2) the satisfaction of male teachers is significantly higher than that of female teachers, there is no significant difference in the overall satisfaction of teachers of different age groups, different degrees, and different titles, and the overall satisfaction of teachers who have worked for more than 10 years is significantly lower than that of teachers with other working years; and (3) there are significant differences in overall satisfaction among students of different gender, family, major, and academic performance. Third, in terms of the most satisfactory and unsatisfactory indicators of teachers and students (see Tables 3, 4), teachers in double world-class universities are most satisfied with the organizational leadership and teaching management measures of the school in entrepreneurship education and most dissatisfied with the lack of professional human resource management strategies for entrepreneurship education teachers. Students in double world-class universities are most satisfied with entrepreneurship policy and least satisfied with entrepreneurship learning, especially in the aspect of entrepreneurship theory learning and practice learning being closely combined with students’ majors. Fourth, in terms of specific factors affecting the overall satisfaction of teachers and students (see Tables 5, 6), the overall satisfaction of students in double world-class universities on the quality of entrepreneurship education mainly comes from the influence of “entrepreneurship policy dividend.” The overall satisfaction degree of teachers in double world-class universities is most affected by the satisfaction degree of organizational leadership, followed by the satisfaction degree of mechanism guarantee and teaching management.

Whereas Wyness et al. (2015) offered a novel insight into entrepreneurship educators’ attitudes to sustainability and their approach to it within their curricula, this study finds that students in double world-class universities are least satisfied with entrepreneurship learning, especially entrepreneurship course content and their professional knowledge being closely integrated. Baoshan and Depeng (2017) found that entrepreneurship competition and awards and entrepreneurial activity experience will affect the students’ entrepreneurial attitude. This study proves this in more depth and finds that students in double world-class universities are most satisfied with entrepreneurship policy.

## Conclusion

The findings of this study give a valuable insight into how teachers and students perceive the entrepreneurship education offered at a university and how satisfied they are with these offerings. The primary data are derived from 12,269 valid questionnaires in student volume and 1,241 valid questionnaires in teacher volume from top universities in China. Furthermore, five hypothesis tests passed. Hypothesis 6 was partially tested (see Tables 5, 6).

### Implications for Theory

The findings of this study have three main theoretical implications. Firstly, this study introduces the sustainable development model of entrepreneurship education in top Chinese universities through the improvement of satisfaction of entrepreneurship education of teachers and students. Secondly, the conclusions of this study extend the work of Gruber et al. (2010) and Baoshan and Depeng (2017) by using new indicators to measure satisfaction of entrepreneurship education. Finally, this study provides detailed evidence for the positive effects of the entrepreneurship education on teachers’ and students’ satisfaction. Moreover, the differences between teachers’ and students’ satisfaction are analyzed in detail. This study can provide a new perspective for other researchers of entrepreneurship education.

### Implications for Practice

This study also has four important practical implications for policy makers and academic institutions that are concerned with entrepreneurship education.

#### Continuously Monitor the Overall Satisfaction of Teachers and Students and Improve Educational Sustainability Development

At present, there is still a lack of research on the satisfaction of teachers and students in entrepreneurship education and a lack of a more mature measurement scale. Double world-class universities are the benchmark of the development of higher education in China. This study provides a preliminary frame of reference for relevant researchers to measure the satisfaction of teachers and students in entrepreneurship education for sustainable development. On the basis of this study, follow-up researchers can carry out surveys and monitor the overall satisfaction of entrepreneurship education of teachers and students in various universities.

#### Pay Attention to the Satisfaction of Entrepreneurship Education of Special Groups

Equity is an important principle of sustainable development. This study found that the overall satisfaction of male teachers was significantly higher than that of female teachers and that the satisfaction of teachers who had worked for more than 10 years was significantly lower than that of teachers with other working years. Furthermore, there are significant differences among students of different nationalities, parents’ entrepreneurial experience, registered permanent residence, entrepreneurial practice experience in school, major, and academic performance. Therefore, in upgrading mass entrepreneurship and innovation background, the government, college, and society should pay more attention to special groups, such as teachers who have worked for more than 10 years, students coming from rural areas, and students with poor academic performance.

#### Strengthen Organizational Leadership and Explore Professional Human Resource Management Strategies for Teachers

The overall satisfaction degree of teachers of entrepreneurship education in double world-class universities is mainly affected by the satisfaction degree of organizational leadership. What the teachers are most dissatisfied with are the professional title evaluation, recruitment, and performance evaluation. Therefore, colleges and universities should continue to strengthen leadership of the organization, innovate personnel policy as soon as possible, and explore the implementation of professional human resource management strategies, including establishing human resource planning, faculty position analysis (full-time, part-time, etc.), recruitment selection (standards of teachers), performance appraisal, and career development.

#### Encouraging Professional-Based Entrepreneurship Is an Important Way to Improve Students’ Satisfaction With Entrepreneurship Education

At present, entrepreneurship policy and entrepreneurship competition are well received by students in double world-class Chinese universities. What the students are most dissatisfied with is the fact that the entrepreneurship course content, the entrepreneurship practice project, and their professional knowledge are not closely integrated. Furthermore, there is a lack of diversified entrepreneurship education courses and teachers with rich experiences in entrepreneurship education. How to further integrate entrepreneurship courses and entrepreneurship practice projects and how to conduct professional-based entrepreneurship are important ways to improve the quality of entrepreneurship education in universities for sustainable development. We can advocate real co-creation between teachers and students; that is, we should pay more attention to the implementation of entrepreneurial projects, instead of simply asking students to take part in entrepreneurial competitions. As the American entrepreneurship expert Shane (2009) found in his research through the correlation analysis of GDP data, entrepreneurship activity data, and employment data of many countries, a large number of typical start-ups did not generate as many jobs and economic contributions as a small number of high-growth start-ups, so policy makers should stop funding the typical start-ups and focus on the high-growth start-ups; that is, they should encourage start-ups based on professional entrepreneurship or cutting-edge technology entrepreneurship.

## Limitations and Further Research Opportunities

In this study, the indicators of satisfaction are subjective to a certain extent. Subsequent researchers can improve them on the basis of the measurement scale developed in this paper. The conceptual frameworks of entrepreneurship education still need more explicit clarification and more in-depth research. The relationship between the satisfaction of entrepreneurship education and the sustainable development of universities remains to be further explored in different contexts.

## Data Availability Statement

The dataset generated for this study will not be made publicly available. Due to the sensitive nature of the questions asked in this study, study participants were assured data would remain confidential and would not be shared. Requests to access the data should be directed to the corresponding author YH, hyj77777@126.com.

## Ethics Statement

Ethical review and approval was not required for the study on human participants in accordance with the local legislation and institutional requirements. Written informed consent from the (patients/participants or patients/participants legal guardian/next of kin) was not required to participate in this study in accordance with the national legislation and the institutional requirements.

## Author Contributions

YH: conceptualization, funding acquisition, methodology, project administration, supervision, writing – original draft, and writing – review and editing. YH and LL: investigation. YH and LA: formal analysis. All authors contributed to the article and approved the submitted version.

## Conflict of Interest

The authors declare that the research was conducted in the absence of any commercial or financial relationships that could be construed as a potential conflict of interest.
